# Impact of sedentary behavior and emotional support on prenatal psychological distress and birth outcomes during the COVID-19 pandemic

**DOI:** 10.1017/S0033291723000314

**Published:** 2023-10

**Authors:** Alison E. Hipwell, Irene Tung, Phillip Sherlock, Xiaodan Tang, Kim McKee, Monica McGrath, Akram Alshawabkeh, Tracy Bastain, Carrie V. Breton, Whitney Cowell, Dana Dabelea, Cristiane S. Duarte, Anne L. Dunlop, Assiamira Ferrera, Julie B. Herbstman, Christine W. Hockett, Margaret R. Karagas, Kate Keenan, Robert T. Krafty, Catherine Monk, Sara S. Nozadi, Thomas G. O'Connor, Emily Oken, Sarah S. Osmundson, Susan Schantz, Rosalind Wright, Sarah S. Comstock

**Affiliations:** 1 Department of Psychiatry, University of Pittsburgh, Pittsburgh, PA, USA; 2 Department of Psychology, California State University Dominguez Hills, Carson, CA, USA; 3 Department of Medical Social Sciences, Northwestern University, Chicago, IL, USA; 4 Department of Family Medicine, University of Michigan, Ann Arbor, MI, USA; 5 Department of Epidemiology, Johns Hopkins School of Public Health, Baltimore, MD, USA; 6 College of Engineering, Northeastern University, Boston, MA, USA; 7 Department of Population and Public Health Sciences, Keck School of Medicine, University of Southern California, Los Angeles, CA, USA; 8 Department of Pediatrics, Department of Population Health, NYU Grossman School of Medicine, New York, NY, USA; 9 Lifecourse Epidemiology of Adiposity and Diabetes (LEAD) Center, University of Colorado Anschutz Medical Campus, Aurora, CO, USA; 10 Department of Psychiatry, Columbia University, New York, NY, USA; 11 Department of Gynecology & Obstetrics, Emory University School of Medicine, Atlanta, GA, USA; 12 Division of Research, Kaiser Permanente Northern California, Oakland, CA, USA; 13 Department of Environmental Health Sciences, Columbia Mailman School of Public Health, New York, NY, USA; 14 Department of Pediatrics, Avera Research Institute, South Dakota School of Medicine, Vermillion, SD, USA; 15 Department of Epidemiology, Geisel School of Medicine, Dartmouth, Lebanon, NH, USA; 16 Department of Psychiatry and Behavioral Neuroscience, University of Chicago, Chicago, IL, USA; 17 Department of Biostatistics and Bioinformatics, Emory University, Atlanta, GA, USA; 18 Departments of Obstetrics & Gynecology, and Psychiatry, Columbia University Medical Center, New York State Psychiatric Institute, New York, NY, USA; 19 Community Environmental Health Program, Health Sciences Center, University of New Mexico, Albuquerque, NM, USA; 20 Departments of Psychiatry, Psychology, Neuroscience, and Obstetrics and Gynecology, University of Rochester, Rochester, NY, USA; 21 Department of Population Medicine, Harvard Medical School and Harvard Pilgrim Health Care Institute, Boston, MA, USA; 22 Department of Obstetrics and Gynecology, Vanderbilt University Medical Center, Nashville, TN, USA; 23 Beckman Institute for Advanced Science and Technology, Urbana, IL, USA; 24 Icahn School of Medicine at Mount Sinai, New York, NY, USA; 25 Department of Food Science & Human Nutrition, Michigan State University, East Lansing, MI, USA

**Keywords:** Birth outcomes, depression, pandemic, pregnancy, stress

## Abstract

**Background:**

Studies have reported mixed findings regarding the impact of the coronavirus disease 2019 (COVID-19) pandemic on pregnant women and birth outcomes. This study used a quasi-experimental design to account for potential confounding by sociodemographic characteristics.

**Methods:**

Data were drawn from 16 prenatal cohorts participating in the Environmental influences on Child Health Outcomes (ECHO) program. Women exposed to the pandemic (delivered between 12 March 2020 and 30 May 2021) (*n* = 501) were propensity-score matched on maternal age, race and ethnicity, and child assigned sex at birth with 501 women who delivered before 11 March 2020. Participants reported on perceived stress, depressive symptoms, sedentary behavior, and emotional support during pregnancy. Infant gestational age (GA) at birth and birthweight were gathered from medical record abstraction or maternal report.

**Results:**

After adjusting for propensity matching and covariates (maternal education, public assistance, employment status, prepregnancy body mass index), results showed a small effect of pandemic exposure on shorter GA at birth, but no effect on birthweight adjusted for GA. Women who were pregnant during the pandemic reported higher levels of prenatal stress and depressive symptoms, but neither mediated the association between pandemic exposure and GA. Sedentary behavior and emotional support were each associated with prenatal stress and depressive symptoms in opposite directions, but no moderation effects were revealed.

**Conclusions:**

There was no strong evidence for an association between pandemic exposure and adverse birth outcomes. Furthermore, results highlight the importance of reducing maternal sedentary behavior and encouraging emotional support for optimizing maternal health regardless of pandemic conditions.

## Introduction

The coronavirus disease 2019 (COVID-19) pandemic has dramatically impacted families globally, exacerbating existing stressors and racial and socioeconomic inequities across a wide range of psychological and health domains (Purtle, 2020; Tai, Shah, Doubeni, Sia, & Wieland, 2021). Common pandemic stressors include health and economic concerns, social isolation, and restrictions on movement (Ammar et al., 2020; Hall, Laddu, Phillips, Lavie, & Arena, 2021). Epidemiological studies have reported an increased prevalence of pandemic-related psychiatric morbidity and psychological distress in the general population (Lei et al., 2020; Smith et al., 2020) with effects projected to continue beyond the current pandemic (Cullen, Gulati, & Kelly, 2020). Studies have also shown increases in the prevalence of psychological distress among women who were pregnant during the COVID-19 pandemic (Berthelot et al., 2020; King, Feddoes, Kirshenbaum, Humphreys, & Gotlib, 2020; Lebel, MacKinnon, Bagshawe, Tomfohr-Madsen, & Giesbrecht, 2020). These trends are particularly concerning given the large body of literature linking prenatal stress and distress with adverse intrauterine development and birth outcomes, such as preterm birth (PTB, < 37 weeks gestation) and low infant birthweight (LBW, <2500 g) (Harville, Xiong, & Buekens, 2010; Lima et al., 2018; Stein et al., 2014). Although evidence suggests that exposure to stress during pregnancy leads to negative birth outcomes, in part *via* heightened maternal psychological distress (e.g. depressive symptoms) (Glover, 2015), there has been limited opportunity to examine the impact of the pandemic as a stressor on prenatal mental health as most studies have been descriptive in nature.

Globally, there have been inconsistent findings about the effect of the pandemic on rates of PTB and low birthweight (Ashish et al., 2020; Been et al., 2020; Hedermann et al., 2021; Kirchengast & Hartmann, 2021; Matheson et al., 2021; Pasternak et al., 2021; Philip et al., 2020). Similarly, in the United States, some studies have reported overall reductions in PTB (Berghella, Boelig, Roman, Burd, & Anderson, 2020; Harvey et al., 2021) or reductions specific to women of White race or from more advantaged neighborhoods (Lemon, Edwards, & Simhan, 2021) relative to rates before the COVID-19 pandemic onset. Other studies have reported no differences (Greene, Kilpatrick, Wong, Ozimek, & Naqvi, 2020; Handley et al., 2021; Wood et al., 2021) or increased rates of very preterm birth specifically among Hispanic or Latinx women (Main et al., 2021). Results are also equivocal with regard to birthweight, with variable evidence for greater infant birthweight (Kirchengast & Hartmann, 2021; Yang et al., 2021), reduced rates of very low birthweight (Philip et al., 2020), or no change (Chmielewska et al., 2021; Matheson et al., 2021) relative to pandemic exposure.

While partly attributable to geographic differences in the timing and extent of pandemic mitigation measures, these mixed results may also reflect differences in the quality and rigor of study designs. Most birth outcome studies have drawn on electronic records to compare rates of PTB and infant birthweight categories before or during the pandemic. However, record-based studies have limited data on important covariates (e.g. maternal race/ethnicity, socioeconomic status) that are associated with experiences of stress and birth outcomes. Because the pandemic disproportionally affected people of color and individuals in low resourced environments (Maroko, Nash, & Pavilonis, 2020), these studies cannot clarify whether the pandemic itself is a driving factor of health outcomes. Methods such as propensity-score matching, a quasi-experimental approach, enable the risks for birth outcomes conferred by the pandemic to be examined separately from those related to sociodemographic factors.

Individual differences in daily behavior and social interactions could also modify the impact of the pandemic on prenatal distress and subsequent birth outcomes. For some individuals, social isolation, loss of daily routines, and enforced working from home led to increased time in sedentary behaviors (Stockwell et al., 2021). Time spent in sedentary behavior is a known risk factor for poor health outcomes, independent of physical activity levels (Clark et al., 2009; Pate, O'Neill, & Lobelo, 2008), and is also associated bi-directionally with mental health problems and perceived stress (Chekroud et al., 2018). Although pregnant women typically spend more than 50% of their waking hours in sedentary behaviors (Fazzi, Saunders, Linton, Norman, & Reynolds, 2017), evidence for birth outcome risks is unclear (Both, Overvest, Wildhagen, Golding, & Wildschut, 2010; Reid, McNeill, Alderdice, Tully, & Holmes, 2014; Ruifrok et al., 2014). Sedentary behavior may exacerbate the impact of the pandemic on prenatal psychological distress and subsequent birth outcomes.

Studies examining pandemic-related health in pregnant women have focused largely on negative impacts. However, to inform strengths-based preventative care, studies need to identify protective factors that can be readily implemented to improve prenatal health and support positive birth outcomes. One factor broadly linked to stress resilience is perceived social support (Panagioti, Gooding, Taylor, & Tarrier, 2014; Sim, Bowes, & Gardner, 2019), particularly emotional support that has been shown to influence stress physiology during pregnancy and may buffer the effects of stress on health outcomes (Coburn, Gonzales, Luecken, & Crnic, 2016; Nierop, Wirtz, Bratsikas, Zimmermann, & Ehlert, 2008; Tung et al., 2021). Although some evidence suggests that higher levels of prenatal support during the pandemic are associated with less psychological distress (Lebel et al., 2020), no studies to our knowledge have directly investigated emotional support as a psychosocial buffer of pandemic effects.

**Fig. 1.**
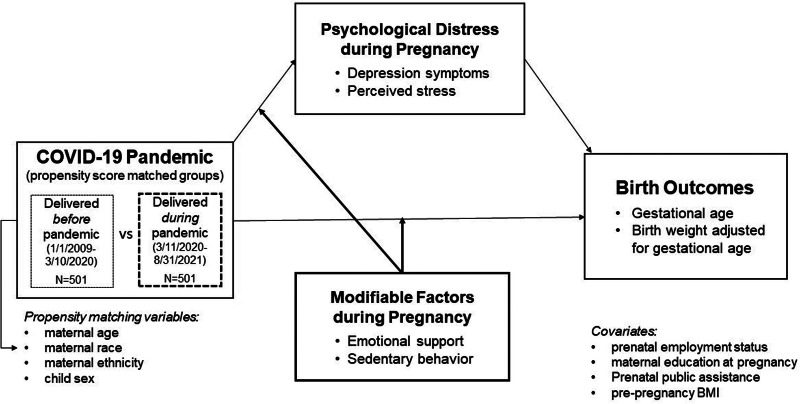
Conceptual model.

Studies have reported mixed findings with respect to the impact of the COVID-19 pandemic on pregnant women and birth outcomes; differences that may be explained in part by sampling, geographical differences and social determinants of health. In the current study, we used propensity-score matching to examine the effects of the pandemic on psychological distress (i.e. perceived stress, depressive symptoms) during pregnancy and on birth outcomes (see conceptual model in Fig. 1). We hypothesized that after controlling for potential sociodemographic confounds, pandemic exposure would be associated with shorter infant gestational age and lower birthweight for gestational age. We also hypothesized that perceived stress and depressive symptoms during pregnancy would mediate the association between pandemic exposure and adverse birth outcomes. Finally, we expected that sedentary behavior would exacerbate, and emotional support would buffer, the negative effects of the pandemic on both prenatal distress and birth outcomes.

## Methods

### Participants

The Environmental influences on Child Health Outcomes (ECHO) Program is an NIH-funded nationwide consortium of multiple cohort studies across the United States designed to investigate the effects of early life exposures on child health and development (Paneth & Monk, 2018; Romano, Buckley, Elliott, Johnson, Paneth, & program collaborators for Environmental influences on Child Health Outcomes, 2022). The ECHO program combines existing prenatal and pediatric data collected via cohort-specific protocols with a standardized ECHO-wide protocol that was established in 2019 (Gillman & Blaisdell, 2018; Knapp et al., in press; LeWinn, Caretta, Davis, Anderson, Oken & program collaborators for Environmental influences on Child Health Outcomes, 2022) (https://echochildren.org/echo-program-protocol/). The ECHO study was approved by the local and/or central ECHO Institutional Review Board, and written informed consent was obtained for participation in specific cohorts and the ECHO-wide data collection protocol.

The current study focused on individuals enrolled in prenatal ECHO cohorts who had a singleton gestation pregnancy and who delivered during or before the COVID-19 pandemic. Between 12 March 2020 and 30 May 2021, 501 pandemic-exposed pregnant women delivered a live infant and had complete sociodemographic data on maternal age, race and ethnicity, and child sex assigned at birth. Given that the decision or ability to participate in research before and during a pandemic may vary for different individuals as a function of sociodemographic characteristics, and evidence that child sex differences can emerge under conditions of stress (Walsh et al., 2019), participants were propensity-score-matched in a 1:1 (pandemic: pre-pandemic) design on the above sociodemographic characteristics with 501 women who delivered before the pandemic onset between 1 January 2009 and 10 March 2020.

### Measures

#### Birth outcomes

Gestational age at birth (GA) in completed weeks and infant birthweight (in grams) were obtained from maternal medical record abstraction (15% GA; 5% birthweight), childbirth/neonatal medical record abstraction (28% GA; 38% birthweight), childbirth information (18% GA; 32% birthweight) or other maternal report (39% GA; 25% birthweight). Sex-specific birthweight adjusted for gestational age *z* scores (BWGA-*z* scores) were assigned based on prior work (Aris, Kleinman, Belfort, Kaimal, & Oken, 2019).

#### Sociodemographic variables

Sociodemographic variables were obtained from maternal medical record abstraction, childbirth/neonatal medical record abstraction, or via maternal report during pregnancy, depending on the ECHO cohort. Maternal age at delivery was calculated from maternal and child dates of birth. Maternal self-reported race was categorized as American Indian or Alaska Native, Asian, Black, White, multiple race, and other race. Self-reported ethnicity was categorized as Hispanic or non-Hispanic. Child sex assigned at birth was coded as female or male.

#### Psychological distress

Severity of prenatal stress was assessed via self-report using the Perceived Stress Scale [PSS, (Cohen, Kamarck, & Mermelstein, 1983). Three versions of the PSS (consisting of 4, 10, or 14 items) were administered across the ECHO cohorts; each item was rated on a 5-point Likert scale. Raw scores were normed to a common, standardized *T* score metric (*Mean* = 50, s.d. = 10) (McDonald, 1999). Maternal depressive symptoms during pregnancy were measured by self-report on at least one of the following: (1) the Patient Reported Outcomes Measurement Information System (PROMIS) Depression 8a (Cella et al., 2010; Pilkonis et al., 2011); (2) the Edinburgh Postnatal Depression Scale (Cox, Holden, & Sagovsky, 1987); (3) the Adult Self-Report Achenbach System Depression Problems Syndrome Scale (Rescorla & Achenbach, 2004); (4) the Brief Symptom Inventory (Derogatis & Melisaratos, 1983); (5) the Center for Epidemiological Studies Depression Scale (Radloff & Locke, 1986); (6) the Patient Health Questionnaire-9 (Kroenke, Spitzer, & Williams, 2001); (7) the Beck Depression Inventory (Beck & Steer, 1984); and (8) the Kessler 6 Mental Health Scale (Kessler et al., 2003). Depression measures were harmonized to the PROMIS *T* score metric using validated crosswalk tables (Blackwell et al., 2021; Cella et al.,; Choi et al., 2022; Kaat, Newcomb, Ryan, & Mustanski, 2017). After harmonization, depression scores were expected to have a mean of 50 and standard deviation (s.d.) of 10 on the PROMIS *T* score (normed for the general population).

#### Sedentary behavior

Sedentary behavior was measured via self-report on the five-item inactivity/sedentary behavior subscale of the Pregnancy Physical Activity Questionnaire (PPAQ), (Chasan-Taber et al., 2004). This PPAQ subscale is a validated and widely used measure for pregnant women (Chasan-Taber et al., 2015; Nascimento, Surita, Godoy, Kasawara, & Morais, 2015).

#### Emotional support

The self-report PROMIS-Emotional Support 4a measure (Cella et al., 2010) assesses the availability of confidante relationships and feeling cared for and valued as a person. PROMIS-Emotional Support 4a applies item response theory to generate *T* scores with scores greater than 50 indicating levels of emotional support higher than in the general population.

### Covariates

Highest level of maternal education reported during pregnancy was reduced to three categories: less than high school, high school completion, or some college and above. Participants reported receipt of any (yes/no) prenatal public assistance (e.g. State Children's Health Insurance Program, Supplemental Nutrition Assistance Program, Head Start, housing assistance, Medicaid, Supplemental Security Income, Temporary Assistance for Needy Families). Prenatal employment status was coded as working or not working for wages. Pre-pregnancy body mass index (BMI) was self-reported or calculated from measured pre-pregnancy weight and height.

### Analytic approach

The study employed a quasi-experimental longitudinal design with women who delivered during or before pandemic onset. Propensity-score matching was used to maximize comparability of the two groups and account for systematic differences in sociodemographic characteristics (i.e. maternal age, maternal race and ethnicity, child sex) based on a 1:1 (pandemic: pre-pandemic) design using the nearest neighbor matching method. A propensity score in the form of probability of belonging to the pandemic or pre-pandemic group conditional on the matching sociodemographic variables was estimated for each case. The pair of cases in the two groups was matched if they had very similar propensity scores (Austin, 2011b). Remaining cases with discrepant propensity scores were removed from the data. Consistent with prior propensity score modeling studies (Austin, 2011a; Rosenbaum & Rubin, 1983), we estimated ‘treatment’ effects (in this case, pandemic exposure) by directly comparing outcomes across matched groups.

We first estimated two multiple regression models with pandemic exposure as the independent variable and infant GA and birthweight for GA *z* score (BWGA-*z* score) as the dependent variables (DV). Models included the following covariates: maternal education level, receipt of public assistance, employment status and pre-pregnancy BMI. We then estimated four structural equation models (SEMs) comprising mediator/DVs as follows: (1) perceived stress/GA; (2) perceived stress/BWGA-*z* score; (3) depressive symptoms/GA; and (4) depressive symptoms/BWGA-*z* score using semTools in the R software package (Jorgensen, Pornprasertmanit, Schoemann, & Rosseel, 2022). Models were conducted in a stepwise fashion to test for the incremental prediction of the predictor and moderator variables. Step 1 tested direct and indirect effects between pandemic exposure, psychological distress (perceived stress or depressive symptoms), and birth outcomes (GA or BWGA-*z* score). In Step 2, the main (direct) effects of sedentary behavior (Step 2a) or emotional support (Step 2b) were added to examine the incremental association effect of these factors on prenatal psychological distress and birth outcomes, above and beyond the effect of the pandemic. Finally, in Step 3, the interactions between sedentary behavior × pandemic (Step 3a) or emotional support × pandemic (Step 3b) were added to examine moderation of the direct effects of pandemic exposure on prenatal psychological distress and birth outcomes. In addition, we used the Index of Moderated Mediation (Hayes, 2015) to test whether sedentary behavior and emotional support moderated the *indirect* associations of pandemic exposure on birth outcomes *via* prenatal psychological distress. The SEM included the following covariates' direct effects on the mediators: education level, receipt of public assistance and employment status, and the same covariates with the addition of pre-pregnancy BMI on birth outcomes. Rates of covariate missingness varied between 4.2% and 31.4% (mean = 13.5%, see Table 1). To minimize parameter biases associated with listwise deletion, missing data on covariates, mediator and moderator variables were imputed using the *mice* package in R (Van Buuren & Groothuis-Oudshoorn, 2011). Multiple imputation (MI) can result in unbiased results with up to 90% missingness with a properly specified MI model that includes all variables related to missingness when data are missing at random (Madley-Dowd, Hughes, Tilling, & Heron, 2019). This resulted in 10 imputed datasets associated with each of the two mediators. The two sets of ten datasets were used to estimate their respective models in *lavaan* (Rosseel, 2012) in R and we reported pooled results.

**Table 1.** Participant characteristics

		Pre-pandemic	Pandemic	Overall	
Variable	Category	(*N* = 501)	(*N* = 501)	(*N* = 1002)	*p* value
Maternal age (years)*					
	Mean (s.d.)	30.9 (5.04)	30.5 (5.01)	30.7 (5.03)	0.161
	Median [min, max]	31.0 [19.0–44.0]	31.0 [19.0–42.0]	31.0 [19.0–44.0]	
	Missing	0	0	0	
Race* *N* (%)					
	White	388 (77.4%)	371 (74.1%)	759 (75.7%)	0.333
	Black	26 (5.19%)	32 (6.39%)	58 (5.79%)	
	Asian	15 (2.99%)	9 (1.80%)	24 (2.40%)	
	American Indian or Alaska Native	12 (2.40%)	17 (3.39%)	29 (2.89%)	
	Multiple Race	36 (7.19%)	50 (9.98%)	86 (8.58%)	
	Other Race	24 (4.79%)	22 (4.39%)	46 (4.59%)	
	Missing	0	0	0	
Ethnicity* *N* (%)					
	Hispanic	121 (24.2%)	141 (28.1%)	262 (26.1%)	
	Non-Hispanic	380 (75.8%)	360 (71.9%)	740 (73.9%)	0.172
	Missing	0	0	0	
Child sex* *N* (%)					
	Female	238 (47.5%)	242 (48.3%)	480 (47.9%)	0.85
	Male	263 (52.5%)	259 (51.7%)	522 (52.1%)	
	Missing	0	0	0	
Educational level *N* (%)					
	Less than high school	20 (4.13%)	16 (3.36%)	36 (3.75%)	0.378
	High school	64 (13.2%)	77 (16.2%)	141 (14.7%)	
	Some college or above	400 (82.6%)	383 (80.5%)	783 (81.6%)	
	Missing	17 (3.4%)	25 (5.0%)	42 (4.2%)	
Prenatal receipt of public assistance *N* (%)					
	No	233 (69.9%)	284 (59.7%)	517 (63.7%)	< 0.001
	Yes	102 (30.4%)	192 (40.3%)	294 (36.3%)	
	Missing	163 (32.7%)	22 (4.4%)	185 (18.6%)	
Income level *N* (%)					
	<$ 30 000	60 (24.8%)	91 (21.7%)	151 (22.8%)	0.366
	$ 30 000–$ 49 999	32 (13.2%)	57 (13.6%)	89 (13.4%)	
	$ 50 000–$ 74 999	36 (14.9%)	68 (16.2%)	104 (15.7%)	
	$ 75 000–$ 99 999	35 (14.5%)	84 (20.0%)	119 (18.0%)	
	$ 100 000 or more	79 (32.6%)	120 (28.6%)	199 (30.1%)	
	Missing	259 (51.7%)	81 (16.2%)	340 (33.9%)	
Prenatal employment for wages, biological mother *N*(%)					
	No (work without pay; homemaker; unemployed)	37 (16.2%)	122 (26.6%)	159 (23.1%)	0.003
	Yes (employed part-time/full-time; self-employed; active duty; on leave and expect to return to work)	191 (83.8%)	337 (73.4%)	528 (76.9%)	
	Missing	273 (54.5%)	42 (8.4%)	315 (31.4%)	
Parity *N* (%)					
	0	176 (45.2%)	34 (13.8%)	210 (33.0%)	< 0.001
	1	121 (31.1%)	134 (54.3%)	255 (40.1%)	
	2	61 (15.7%)	37 (15.0%)	98 (15.4%)	
	3	19 (4.88%)	21 (8.50%)	40 (6.29%)	
	⩾	12 (3.08%)	21 (8.50%)	33 (5.19%)	
	Missing	112 (22.4%)	254 (50.7%)	366 (36.5%)	
Gestational age at birth (weeks)					
	Mean (s.d.)	39.0 (1.88)	38.7 (1.75)	38.8 (1.83)	0.002
	Median [min, max]	39.0 [23.0–42.0]	39.0 [23.0–43.0]	39.0 [23.0–43.0]	
	Missing	<5	8 (1.6%)	8 (0.8%)	
Gestational age category *N* (%)					
	Extremely and very preterm (22–33 weeks)	<10	<10	13 (1.31%)	<0.001
	Late preterm (34–36 weeks)	24 (4.79%)	26 (5.27%)	50 (5.03%)	
	Early term (37–38 weeks)	100 (20.0%)	153 (31.0%)	253 (25.5%)	
	Full term (39–40 weeks)	299 (59.7%)	267 (54.2%)	566 (56.9%)	
	Late term (> 41 weeks)	71 (14.2%)	41 (8.32%)	112 (11.3%)	
	Missing	<5	<10	8 (0.8%)	
Birthweight (grams)					
	Mean (s.d.)	3370 (537)	3400 (508)	3380 (525)	0.507
	Median [min, max]	3390 [600–4930]	3430 [539–4620]	3400 [539–4930]	
	Missing	20 (4.0%)	164 (32.7%)	184 (18.4%)	
Birthweight category *N* (%)					
	Low birthweight (<2500 g)	24 (4.99%)	12 (3.56%)	36 (4.40%)	0.172
	Normal birthweight (⩾2500 g and <4000 g)	402 (83.6%)	297 (88.1%)	699 (85.5%)	
	Macrosomia (⩾4000 g and <5000 g)	50 (10.4%)	27 (8.1%)	83 (10.2%)	
	Missing	<25	<170	184 (18.4%)	
Birthweight for GA *z* score (BWGA-*z* score)					
	Mean (s.d.)	0.0174 (1.06)	0.234 (0.960)	0.107 (1.02)	0.002
	Median [min, max]	0.00858 [−3.04 to 3.08]	0.256 [−2.37 to 2.54]	0.131 [−3.04 to 3.08]	
	Missing	20 (4.0%)	164 (32.7%)	184 (18.4%)	
Pre-pregnancy BMI					
	Mean (s.d.)	26.6 (6.61)	28.1 (7.26)	27.2 (6.94)	0.002
	Median [min, max]	24.8 [16.8–60.4]	26.9 [13.9–67.7]	25.6 [13.9–67.7]	
	Missing	16 (3.2%)	112 (22.4%)	128 (12.8%)	
Perceived stress harmonized *T* score					
	Mean (s.d.)	48.3 (9.54)	47.1 (10.1)	47.7 (9.83)	0.064
	Median [min, max]	48.5 [22.4–72.5]	46.6 [22.4–78.2]	47.3 [22.4–78.2]	
	Missing	31 (6.2%)	15 (3.0%)	46 (4.6%)	
Depressive symptoms harmonized *T* score					
	Mean (s.d.)	46.0 (8.61)	48.0 (8.50)	46.9 (8.61)	0.003
	Median [min, max]	45.9 [33.0–81.8]	47.8 [33.0–71.6]	45.9 [33.0–81.8]	
	Missing	136 (27.1%)	194 (38.7%)	330 (32.9%)	
PPAQ Sedentary subscale					
	Mean (s.d.)	15.6 (9.46)	14.6 (7.29)	15.3 (8.75)	0.11
	Median [min, max]	13.9 [0–46.7]	14.5 [0–39.8]	14.2 [0–46.7]	
	Missing	29 (5.8%)	232 (46.3%)	261 (26.0%)	
PROMIS emotional support 4a *T* score					
	Mean (s.d.)	57.6 (6.95)	57.5 (6.92)	57.5 (6.92)	0.93
	Median [min, max]	62.0 [36.9–62.0]	62.0 [30.4–62.0]	62.0 [30.4–62.0]	
	Missing	431 (86.0%)	147 (29.3%)	578 (57.7%)	

*BMI*, body mass index; *BWGA,* birthweight for gestational age; *GA,* gestational age; *max*, maximum; *min*, minimum; *PPAQ*, Pregnancy Physical Activity Questionnaire; *PROMIS*, Patient Reported Outcomes Measurement Information System; s.d., standard deviation.

Note. Cell sizes smaller than 5 are suppressed for privacy in accordance with ECHO's publication and data use policy. Variables with* were covariates used in propensity score matching. Complete data of these variables were available

Groups were compared using *t* tests for continuous variables. For categorical variables, *p* values for χ^2^ tests were computed across categories excluding the missing category between the pre-pandemic and pandemic groups

## Results

### Descriptive Statistics

Sample characteristics are shown in Table 1. In the overall sample (*N* = 1002, drawn from 16 ECHO cohorts, see online Supplementary Table S1), participants were on average 30.7 years old (s.d. = 5.03). Most women self-identified as White (75.7%) with 5.8% as Black, 2.4% as Asian, 2.9% as American Indian or Alaska Native, 8.6% more than one race and 4.6% another race, and most participants reported non-Hispanic ethnicity (73.9%). Infants (47.9% female) had an average GA of 38.8 weeks (s.d. = 1.83); 6.3% were born preterm (<37 weeks) and mean birthweight was 3380 g (s.d. = 525). GA was unrelated to BWGA-*z* score (*r* = −0.01, *ns*) indicating their independence for later model estimation. Mean harmonized perceived stress and depression *T* scores for the overall sample were 47.7 (s.d. = 9.83) and 46.9 (s.d. = 8.61) respectively, close to the population norm. Approximately half of the participants in the exposed group (*n* = 261, 52%) became pregnant after the start of the pandemic, whereas 97 participants (19.4%) were in the third trimester.

By design, the pandemic and pre-pandemic groups did not differ on maternal age, race, ethnicity and child sex. There was also no group difference on education or income level. However, relative to women in the pre-pandemic group, pandemic-exposed women were more likely to receive public assistance, less likely to be employed and had higher pre-pregnancy BMI (*p*s < 0.01). These variables were covaried in the predictive and mediation models to account for these group differences.

### Effects of pandemic exposure on birth outcomes

Results of the multiple regression models after controlling for covariates showed a small effect of prenatal pandemic exposure on shorter GA at birth [*β* = −0.56 weeks, 95% CI (−0.89 to −0.24)]. In contrast, pandemic exposure was unrelated to adjusted birthweight [*β* = 0.01, 95% CI (−0.17 to 0.20)]. Maternal education, receipt of public assistance, and employment status were not significantly associated with birth outcomes.

### Mediation models

#### Pandemic, perceived stress and GA at birth

As shown in Table 2, after adjusting for covariates in Step 1, women who were pregnant during the pandemic reported higher levels of stress compared to those who were pregnant pre-pandemic [*B* = 2.53, standard error (s.e.) = 0.99, 95% CI (0.59–4.47)]. Furthermore, after adjusting for covariates and perceived stress during pregnancy, pandemic exposure had a small direct effect on GA at birth (*B* = −0.55, s.e. = 0.18): between 0.20 and 0.90 weeks shorter than pre-pandemic births. However, prenatal stress did not mediate the association between pandemic exposure and infant GA. Higher levels of sedentary behavior were associated with higher levels of perceived stress beyond the significant effects of pandemic status and public assistance (Table 2 GA; Step 2a), but no main effect of sedentary behavior on GA at birth was observed. In Step 2b, higher levels of emotional support were associated with less perceived stress but did not directly predict GA at birth. Neither sedentary behavior nor emotional support moderated the direct and indirect effects of the pandemic on perceived stress and infant GA at birth (Steps 3a and 3b, results not shown).

**Table 2.** Structural equation models examining perceived stress as mediating the effect of pandemic on birth outcomes

				Gestational age at birth		
Perceived stress	*B*	s.e.	95% CI [min, max]	*B*	s.e.	95% CI [min, max]
Step 1						
Pandemic exposure	**2.53**	**0.99**	**0.590–4.470**	**−0.55**	**0.18**	**−0.903 to −0.197**
Perceived stress	NA	NA	NA	−0.004	0.01	−0.024 to 0.016
Education	−1.13	1.06	−3.208 to 0.948	−0.11	0.20	−0.502 to 0.282
Public assistance	1.55	1.05	−0.051 to 3.608	0.05	0.19	−0.322 to 0.422
Employment	−1.52	1.12	−3.715 to 0.675	−0.18	0.21	−0.592 to 0.232
BMI	NA	NA	NA	**−0.03**	**0.01**	**−0.050 to −0.010**
Step 2a						
Pandemic exposure	**4.28**	**1.09**	**2.144–6.416**	−0.61	0.22	−1.041 to 0.179
Perceived Stress	NA	NA	NA	−0.004	0.01	−0.024 to 0.016
Education	−0.57	1.18	−2.883 to 1.743	0.03	0.24	−0.440 to 0.500
Public assistance	**2.52**	**1.18**	**0.207–4.833**	0.14	0.24	−0.330 to 0.610
Employment	−1.92	1.34	−4.546 to 0.706	−0.39	0.27	−0.919 to 0.139
BMI	NA	NA	NA	**−0.03**	**0.01**	**−0.050 to −0.010**
Sedentary behavior	**0.19**	**0.05**	**0.092–0.288**	−0.002	0.01	−0.022 to 0.018
Step 2b						
Pandemic exposure	0.94	1.43	−1.863 to 3.743	−0.01	0.29	−0.578 to 0.558
Perceived stress	NA	NA	NA	−0.01	0.01	−0.030 to 0.010
Education	0.16	1.11	−2.016 to 2.336	−0.22	0.22	−0.651 to 0.211
Public assistance	1.36	1.02	−0.639 to 3.359	−0.04	0.21	−0.452 to 0.372
Employment	−0.31	1.19	−2.642 to 2.022	−0.24	0.24	−0.710 to 0.230
BMI	NA	NA	NA	**−0.03**	**0.01**	**−0.050 to −0.010**
Emotional support	**−0.62**	**0.07**	**−0.757 to −0.483**	0.01	0.02	−0.029 to 0.049

				Birthweight for gestational age *z* score		
Perceived stress	*B*	s.e.	95% CI [min, max]	*B*	s.e.	95% CI [min, max]
Step 1						
Pandemic exposure	**2.57**	**1.05**	**0.512–4.628**	0.01	0.1	−0.186 to 0.206
Perceived stress	NA	NA	NA	−0.01	0.01	−0.030 to 0.010
Education	−0.72	1.12	−2.895 to 1.475	−0.07	0.11	−0.286 to 0.146
Public assistance	1.71	1.13	−0.505 to 3.925	0.07	0.11	−0.146 to 0.286
Employment	−0.56	1.21	−2.932 to 1.812	0.01	0.12	−0.225 to 0.245
BMI	NA	NA	NA	0.01	0.01	0–0.030
Step 2a						
Pandemic exposure	**4.15**	**1.11**	**1.974–6.326**	0.06	0.11	−0.156 to 0.276
Perceived stress	NA	NA	NA	−0.01	0.01	−0.030 to 0.010
Education	−0.48	1.18	−2.793 to 1.833	−0.13	0.12	−0.365 to 0.105
Public assistance	**2.62**	**1.22**	**0.229–5.011**	0.07	0.12	−0.165 to 0.305
Employment	−1.76	1.35	−4.406 to 0.886	−0.04	0.14	−0.314 to 0.234
BMI	NA	NA	NA	**0.02**	**0.01**	**0.001–0.040**
Sedentary behavior	**0.19**	**0.05**	**0.092–0.288**	−0.000	0.01	−0.020 to 0.020
Step 2b						
Pandemic exposure	0.012	1.51	−2.948 to 2.972	−0.01	0.16	−0.324 to 0.304
Perceived stress	NA	NA	NA	−0.01	0.01	−0.030 to 0
Education	0.27	1.14	−1.964 to 2.504	−0.05	0.12	−0.285 to 0.185
Public assistance	1.45	1.07	−0.647 to 3.547	0.14	0.11	−0.076 to 0.356
Employment	0.33	1.24	−2.100 to 2.760	0.15	0.13	−0.105 to 0.405
BMI	NA	NA	NA	0.01	0.01	−0.010 to 0.030
Emotional support	**−0.60**	**0.08**	**−0.757** to **−0.443**	−0.02	0.01	−0.040 to 0

*B,* unstandardized beta; *BMI*, body mass index; *CI*, confidence interval; *max*, maximum; *min*, minimum; *NA*, not applicable; s.e., standard error.

*Note*: Significant effects are bolded for emphasis.

#### Pandemic, perceived stress and BWGA

In adjusted models, pandemic exposure showed no direct effect on offspring BWGA-*z* score (Table 2 BWGA; Step 1). Additionally, there was no main effect of sedentary behavior on adjusted birthweight after accounting for sociodemographic and health covariates, including the significant effects of pre-pregnancy BMI (Table 2 BWGA; Step 2a). Similarly, emotional support did not directly predict BWGA-*z* score (Step 2b). Neither sedentary behavior nor emotional support moderated direct or indirect effects of the pandemic (Steps 3a and 3b, results not shown).

#### Pandemic, depressive symptoms and GA at birth

Models examining depressive symptoms as mediating the effect of the pandemic on birth outcomes are shown in Table 3. In Step 1, pandemic-exposed women reported higher levels of prenatal depressive symptoms [*B* = 3.12, s.e. = 1.07, 95% CI (1.02–5.22)] after adjusting for covariates. In addition, infants delivered during the pandemic had somewhat shorter GA at birth compared to infants delivered pre-pandemic [*B* = −0.71, s.e. = 0.25, 95% CI (−1.20 to −0.22)]. However, prenatal depressive symptoms did not predict variability in GA, nor did they mediate the association between pandemic exposure and GA at birth. More sedentary behavior was associated with higher levels of prenatal depressive symptoms over and above the significant effects of pandemic status (Table 3 GA; Step 2a). However, no main effect of sedentary behavior on infant GA was observed beyond the significant effect of pandemic status and adjustment for covariates. In Step 2b, emotional support was uniquely associated with lower levels of prenatal depressive symptoms but was unrelated to GA at birth. After adjusting for emotional support, pandemic exposure remained significantly associated with higher prenatal depressive symptoms, although it no longer predicted shorter GA at birth. Neither emotional support nor sedentary behavior moderated the direct and indirect effects of the pandemic on prenatal depressive symptoms and infant GA (Steps 3a and 3b, results not shown).

**Table 3.** Structural equation models examining depressive symptoms as mediating the effect of the pandemic on birth outcomes

	Depressive symptoms			Gestational age at birth		
	*B*	s.e.	95% CI [min, max]	*B*	s.e.	95% CI [min, max]
Step 1						
Pandemic exposure	**3.12**	**1.07**	**1.023–5.217**	**−0.71**	**0.25**	**−1.200to −0.220**
Depressive symptoms	NA	NA	NA	−0.01	0.01	−0.030 to 0.010
Education	−0.91	1	−2.870 to 1.050	−0.02	0.23	−0.471 to 0.431
Public assistance	**2.08**	**1**	**0.120–4.040**	0.06	0.23	−0.391 to 0.511
Employment	0.47	1.1	−1.686 to 2.626	−0.48	0.25	−0.970 to 0.010
BMI	NA	NA	NA	**−0.02**	**0.01**	**−0.040 to −0.001**
Step 2a						
Pandemic exposure	**3.63**	**1.03**	**1.611–5.649**	**−0.76**	**0.27**	**−1.289 to −0.231**
Depressive symptoms	NA	NA	NA	−0.01	0.01	−0.030 to 0.010
Education	−1.29	1.06	−3.367 to 0.788	0.01	0.27	−0.519 to 0.539
Public assistance	**2.64**	**0.99**	**0.700–4.580**	0.10	0.26	−0.410 to 0.610
Employment	−0.79	1.19	−3.122 to 1.542	−0.53	0.30	−1.118 to 0.058
BMI	NA	NA	NA	−0.02	0.02	−0.059 to 0.019
Sedentary behavior	**0.22**	**0.07**	**0.083–0.357**	0.001	0.02	−0.038 to 0.040
Step 2b						
Pandemic exposure	**3.23**	**1.51**	**0.270–6.190**	0.17	0.36	−0.536 to 0.876
Depressive symptoms	NA	NA	NA	−0.02	0.01	−0.043 to 0.011
Education	−0.16	1.01	−2.140 to 1.820	−0.18	0.24	−0.650 to 0.290
Public assistance	1.29	0.97	−0.611 to 3.191	0.09	0.23	−0.361 to 0.541
Employment	1.69	1.14	−0.544 to 3.924	−0.37	0.27	−0.899 to 0.159
BMI	NA	NA	NA	−0.03	0.02	−0.069 to 0.010
Emotional support	**−0.43**	**0.07**	**−0.567 to −0.293**	0.003	0.02	−0.036 to 0.042

	Depressive symptoms			Birthweight for gestational age *z* score		
	*B*	s.e.	95% CI [min, max]	*B*	s.e.	95% CI [min, max]
Step 1						
Pandemic exposure	**2.80**	**1.11**	**0.624–4.976**	0.03	0.13	−0.225 to 0.285
Depressive symptoms	NA	NA	NA	−0.002	0.01	−0.022 to 0.018
Education	−0.74	1.01	−2.720 to 1.240	0.000	0.12	−0.235 to 0.235
Public assistance	**2.06**	**1.03**	**0.041–4.079**	0.12	0.12	−0.115 to 0.355
Employment	0.48	1.12	−1.715 to 2.675	0.02	0.13	−0.235 to 0.275
BMI	NA	NA	NA	0.01	0.01	−0.010 to 0.030
Step 2a						
Pandemic exposure	**3.42**	**1.06**	**1.342–5.498**	0.09	0.14	−0.184 to 0.364
Depressive symptoms	NA	NA	NA	−0.01	0.01	−0.030 to 0.010
Education	−1.18	1.07	−3.28 to 0.917	0.000	0.14	−0.274 to 0.274
Public assistance	**2.66**	**1.02**	**0.661–4.659**	0.10	0.13	−0.155 to 0.355
Employment	−0.61	1.20	−2.962 to 1.742	0.01	0.15	−0.284 to 0.304
BMI	NA	NA	NA	0.01	0.01	−0.010 to 0.030
Sedentary behavior	**0.22**	**0.07**	**0.083–0.357**	0.002	0.01	−0.018 to 0.022
Step 2b						
Pandemic exposure	2.51	1.63	−0.685 to 5.705	−0.04	0.20	−0.432 to 0.352
Depressive symptoms	NA	NA	NA	0.00	0.01	−0.020 to 0.020
Education	0.09	1.03	−1.929 to 2.109	−0.13	0.13	−0.385 to 0.125
Public assistance	1.20	1.01	−0.780 to 3.180	0.14	0.12	−0.095 to 0.375
Employment	1.65	1.15	−0.604 to 3.904	0.09	0.14	−0.184 to 0.364
BMI	NA	NA	NA	0.01	0.01	−0.010 to 0.030
Emotional support	**−0.43**	**0.07**	**−0.567 to −0.293**	−0.01	0.01	−0.030 to 0.010

*B,* unstandardized beta; *BMI*, body mass index; *CI*, confidence interval; *max*, maximum; *min*, minimum; *NA*, not applicable; s.e., standard error.

*Note*: Significant effects are bolded for emphasis.

#### Pandemic, depressive symptoms and BWGA

In adjusted models, neither pandemic exposure, nor prenatal depressive symptoms, predicted offspring BWGA-*z* score (Table 3 BWGA; Step 1). In Step 2a, sedentary behavior was associated with higher depressive symptoms beyond the significant effects of pandemic status, whereas in Step 2b, emotional support was associated with fewer depressive symptoms. BWGA-*z* score was unrelated to sedentary behavior or emotional support in adjusted models. Sedentary behavior and emotional support did not moderate any direct or indirect effects of the pandemic (Steps 3a and 3b).

## Discussion

There is an urgent need for rigorously designed studies to examine the impact of the pandemic on women's prenatal health and subsequent birth outcomes, as well as studies that can identify modifiable daily life factors that could exacerbate or attenuate pandemic effects. The ECHO study provides a valuable opportunity to fill these gaps via common data elements collected before and during the pandemic from cohorts located across the United States. The current study used propensity-score matching to increase causal inferences made about the effect of the pandemic on birth outcomes and determine whether heightened psychological distress associated with the pandemic explained these effects.

The results showed that women pregnant during the pandemic reported higher levels of stress and depressive symptoms compared with a propensity-score matched group of women who delivered prior to the pandemic. This increase may reflect the disruptions to daily life and health, social, and financial concerns experienced by many during the pandemic (Fitzpatrick, Drawve, & Harris, 2020; Tai et al., 2021), and is consistent with prior descriptive studies showing increased prevalence of psychiatric disorders and psychological distress. However, by leveraging a quasi-experimental design, the current study could increase the sociodemographic comparability of the pandemic and pre-pandemic groups to provide a more rigorous test of exposure on prenatal distress. This approach, combined with inclusion of additional covariates, allowed us to delineate the effects of the pandemic from the effects of various sociodemographic confounders.

Contrary to our hypothesis, the study did not reveal a substantial negative effect of pandemic exposure on birth outcomes. Although the results showed a shorter GA in the pandemic relative to the pre-pandemic group, this effect translated to an overall mean difference of about half a week, which may be important for preterm births, but may have little clinical significance for early term and term births. This result is commensurate with several other U.S.-based studies that have shown no, or only a small association, between pandemic exposure and categorical definitions of preterm birth (Greene et al., 2020; Handley et al., 2021; Wood et al., 2021). In addition, the current study revealed no main effects of the pandemic on GA-adjusted infant birthweight, similar to some prior descriptive studies focused on (unadjusted) birthweight (Chmielewska et al., 2021) but at odds with others conducted outside the United States (Yang et al., 2021). Taken together, our results suggest that pandemic mitigation measures (e.g. focus on hygiene, physical distancing, reduced physical demands of work and travel) while not reducing psychological distress, may have been generally effective in protecting some women' (Goldenberg, Culhane, Iams, & Romero, 2008).

Despite the elevated rates of psychological distress among women pregnant during the pandemic, neither perceived stress nor depressive symptoms predicted birth outcomes beyond the effect of the pandemic. Thus, our hypothesis that psychological distress would mediate the association between prenatal pandemic exposure and negative birth outcomes was not supported. However, this study may only partially capture the range of stress and depression, or birth outcomes experienced by pregnant women in the United States. Specifically, most participants in the analytic sample identified as White (75.7%), non-Hispanic (73.9%), and college educated (81.6%), whereas other racial and ethnic groups and individuals with fewer resources were under-represented. Thus, our observation of minimal effects of the pandemic on birth outcomes may be most relevant to highly educated White women in the United States; an important consideration given that systemic racism and structural processes underlying economic disparities significantly contribute to known inequities in prenatal stress and birth outcomes (Alhusen, Bower, Epstein, & Sharps, 2016; Braveman et al., 2015; Mendez, Hogan, & Culhane, 2013). Given the elevated rates of preterm birth among Black, American Indian, and Hispanic/Latinx infants (March of Dimes Foundation, 2022), there is a clear need for additional studies that focus specifically on the impact of the pandemic on prenatal distress and birth outcomes for these groups. Future work should also consider the contributions of psychological resources, given evidence that resilience, optimism and life satisfaction are associated with offspring birth outcomes (Bhatia, Chao, Higgins, Patel, & Crespi, 2015; Maxson, Edwards, Valentiner, & Miranda, 2016) and may explain variability beyond prenatal distress (Ramiro-Cortijo et al., 2021). Thus, it is possible that the current findings masked subgroups differentially characterized by personal resources.

An important strength of the current study was examination of potential pandemic-related effect modifiers (sedentary behavior and emotional support) with relevance for health policy and practice. The results showed a consistent pattern of main effects, whereby sedentary behavior was associated with higher levels of perceived stress and depressive symptoms, and emotional support was robustly associated with lower levels of each. However, none of the hypothesized moderating effects were observed. Although sedentary behavior did not exacerbate the negative effects of the pandemic on distress or birth outcomes in the current analysis, the additive risk to psychological distress highlights a universal need for targeted interventions that reduce sedentary behavior to improve psychological health during pregnancy (DiPietro et al., 2019; Kołomańska, Zarawski, & Mazur-Bialy, 2019), regardless of pandemic conditions. Furthermore, despite a lack of association with birth outcomes in the current study, sedentary behavior likely confers risk for maternal cardiovascular diseases such as hypertension, diabetes, and metabolic syndrome (Narici et al., 2021) that could impact the health of future pregnancies (Xie, Madkour, & Harville, 2015).

Emotional support was robustly associated with lower levels of prenatal stress and depressive symptoms and, in most cases, the negative effect of the pandemic on psychological distress became negligible once emotional support was accounted for. These results support the utility of emotional support as a critical target for healthcare efforts in terms of both screening and intervention (Dunkel Schetter, 2011; Marques, Bjørke-Monsen, Teixeira, & Silverman, 2015). Emotional support can take many forms such as having a confidante, friends and family in the community, connections with health workers (Hans, Edwards, & Zhang, 2018; Orr, 2004), and/or perinatal support groups (Chae, Chae, Kandula, & Winter, 2017; Chan & Chen, 2019). Further research is needed to understand how pregnant women best access/receive emotional support, and the types that are most impactful on psychological well-being during pregnancy.

### Limitations

The findings should be considered in the context of several limitations. First, given some constraints on the availability of data, propensity-score matching of the pandemic and pre-pandemic groups was limited to four sociodemographic variables. Although the groups were comparable on educational level and income level, and all women had a singleton pregnancy, descriptive data indicated that some important differences remained on variables including receipt of public assistance, paid employment, and pre-pregnancy BMI. In addition, limited data on parity prevented inclusion of this variable in analyses. Given associations with birth outcomes, including PTB (Koullali et al., 2020), this is an important covariate for future studies. Unmeasured cohort or period effects (e.g. political climate, population health, mental health awareness) could have affected outcomes. Second, data were gathered from a 15-month period during the pandemic (11 March 2020 to 30 May 2021) during which infection rates and mitigation measures varied. While this extended interval fully captured the entire pregnancy for more than half the women unlike some prior studies, there was likely a range in the type, duration, and severity of stress experienced by women (e.g. disruptions to prenatal health care, risk for infection, social isolation, job loss) as well as differences in local and state-level mitigation policies at varying times across the pregnancy that were not modeled. Future studies are needed to examine more fine-grained pandemic experiences in relation to birth outcomes, and to capture the full range of pregnancy experiences and birth outcomes in diverse groups of women. Finally, sample bias may have been introduced by the focus on GA and birthweight among live births included in the ECHO study given some evidence suggesting a higher incidence of stillbirths during the pandemic (Khalil et al., 2020).

## Conclusion

Using a quasi-experimental design, our results showed that exposure to the COVID-19 pandemic during pregnancy was associated with heightened psychological distress during pregnancy and marginally shorter GA at birth. In addition, we observed a general, but not a pandemic-specific, effect of sedentary behavior and emotional support on prenatal stress and depressive symptoms, highlighting the importance of these factors for maternal health regardless of pandemic exposure.

## Supplementary material

For supplementary material accompanying this paper visit https://doi.org/10.1017/S0033291723000314. click here to view supplementary material
