# Supplementary material for: Impact of sedentary behavior and emotional support on prenatal psychological distress and birth outcomes during the COVID-19 pandemic
Source: Psychol Med. 2023 Mar 8;53(14):6792–805. doi: 10.1017/S0033291723000314 (PMC10485176; doi:10.1017/S0033291723000314)
Supplement: Supplementary file 1 [file S0033291723000314sup001.docx]

**Supplementary Table S1. ECHO contributing cohorts**

| **Collection Start Date** | **Cohort Name** | **Sample Description** | **Study Aims** | **Inclusion/Exclusion** | **Analytic sample n** |
| --- | --- | --- | --- | --- | --- |
| 2011 | University of Puerto Rico (PROTECT) | Pregnant women recruited from 5 OB/GYN clinics in Puerto Rico and their offspring. | To understand environmental exposures and their effects on birth outcomes and children’s neurodevelopmental health in Puerto Rico. | Pregnant woman with a gestational age of less than 20 weeks; Planned to give birth in one of the affiliated hospitals; Seeking prenatal care in one of the affiliated clinics. / Age (less than 18 or over 40 yrs would be excluded); Multi fetal pregnancies; Have used contraceptive methods on the 3 months prior pregnancy; Have used in vitro fertilization to conceive; History of threatened abortion; Major vaginal bleeding; Cardiac Disease prior to pregnancy; Chronic high blood pressure; History of neuropathy; Liver disease prior to pregnancy | 73 |
| 2009 | Healthy Start | Colorado community sample of mother-child dyads recruited in infancy from obstetrics clinics at a university hospital and by word of mouth, as well as medical university employees. | To understand the contribution of metabolic and behavioral factors during pregnancy to the development of childhood obesity, insulin resistance, and inflammatory markers. | Women with a singleton pregnancy; Less than or equal to 23 weeks gestation; Age greater than or equal to 16 years. / Cancer; Psychiatric diseases; steroid-dependent asthma; pre-existent diabetes mellitus; previous premature delivery; prior low birth; subsequent fetal death of index child | 220 |
| 2011 | PRogramming of Intergenerational Stress Mechanisms (PRISM) | Pregnant women recruited from prenatal clinics at hospitals and community health centers in Boston, MA and New York, NY, and their offspring. | To examine how prenatal and childhood chemical and non-chemical pro-oxidant environmental factors impact child neurodevelopment, stress regulation, and respiratory health. | Mother 18 years or older at recruitment in pregnancy; Single gestation pregnancy; Mother English or Spanish speaking. / At enrollment, endorsement of drinking >= 7 alcoholic drinks/week prior to pregnancy recognition; At enrollment, endorsement of any drinking after pregnancy recognition; Maternal or child chronic health conditions that would impede study participation | 32 |
| 2015 | Maternal and Infant Nutrition, Neurodevelopment, and In utero Exposures (MINNIE) | Children recruited between 3 months to 12 years of age from the general population recruited in pediatrician offices and via online webpages, radio advertisements, and flyers in Providence, RI. | To examine typical brain development, including how brain growth is altered by specific pre- and post-natal environmental or genetic factors; how patterns of brain growth are associated with, and predictive of, emerging cognitive and behavioral abilities; and how these brain-behavior relationships are influenced by modifiable factors experienced throughout childhood. | Pregnant mothers with uncomplicated pregnancies; No Alcohol, cigarette or illicit substance use (including marijuana); Singleton pregnancy; No abnormalities on first trimester fetal ultrasound; No major psychiatric disorder (including depression requiring medication); Preterm (<37w) birth; No NICU admission; No history of neurological disorder (e.g., head injury, epilepsy) in infant; No history of psychiatric or learning disorder in the infant, parents, or siblings. / NA | 49 |
| 2000 | Boricua Youth Study (BYS) | Latinx youth identifying as being of Puerto Rican background recruited in New York, NY and Puerto Rico during childhood and their caregivers. | To examine key factors that influence intergenerational socioeconomic disadvantage and the impact of adversity on child neurodevelopmental health and functioning. | Participation in Boricua Youth Study, Wave 1, which required: Being between the ages of 5–13 in 2001-2002; Living in South Bronx, New York or San Juan Metropolitan Area and Caguas, Puerto Rico in 2001-2002; Identification of a youth as being of Puerto Rican background at enumeration of BYS W1; At least one of the youth's caretakers identified as Puerto Rican background. / Youth known to be severely impaired neuropsychologically; Youth who are permanently institutionalized or youth who have run away or left home; Emancipated youth whose parent cannot provide consent | 18 |
| 2014 | Atlanta ECHO Cohort of EMORY University | African American women recruited from two prenatal clinics in Atlanta, GA and their offspring. | To identify intrauterine and early childhood environmental exposures and risk pathways contributing to children’s neurodevelopmental deficits and obesity. | African American (self-identified as Black/African American and self-report as US-born); 18-40 years of age at the time of enrollment; Singleton pregnancy between 8-14 weeks; Fewer than 5 previous births; Without chronic medical conditions and not taking prescription medications chronically. / Post-enrollment: Intrauterine death; Post-enrollment: Congenital anomalies | 9 |
| 2016 | Safe Passage Study (PASS) | White and American Indian women recruited in pregnancy from participating obstetrics and gynecology clinics and other entities (e.g., the Supplemental Nutrition Program for Women, Infants, and Children) in South Dakota. | To investigate the relationship between prenatal alcohol exposure, stillbirth, and sudden infant death syndrome. | Pregnant female of any race or ethnicity carrying one or two fetuses during pregnancy; Age 16 years or older, inclusive at time of consent; At time of recruitment visit, participant is at least 6 weeks, 0 days and <20 weeks, 1 day gestation; con't from 3 - OR participant is 20 weeks, 1 day gestation, has not had more than 2 prenatal visits; con't from 4 - AND the current visit is not the delivery admission; Must speak English (Northern Plains only); Able to provide informed consent. / Women carrying three or more fetuses during the pregnancy; Planned abortion; Moving out of catchment area prior to estimated date of delivery; Unable to provide informed consent; Health care provider advises against participation | 260 |
| 2013 | Pregnancy Environment and Lifestyle Study (PETALS) | Women identified as pregnant via hospital records from 4 California hospitals recruited before 10 weeks gestation via telephone and their offspring. | To examine whether BPA levels are associated with gestational diabetes and growth development. | Pregnant women; Current members at Kaiser Permanente Northern California. / Age <18 years; Pre-existing diabetes diagnosis; Severe liver disease (hepatitis C, cirrhosis); Multiple pregnancy | 109 |
| 2019 | Childhood Allergy and the Neonatal Environment (CANOE) | A diverse multicenter birth cohort of families recruited from Detroit, MI, Madison, WI, Nashville, TN, and St. Louis, MO where all families have at least one parent with allergic disease or asthma. | To identify prenatal and early life exposures that are related to the development of allergies and wheezing illnesses in the first two years of life. | History of or concurrent asthma, allergic rhinitis (hay fever) or atopic dermatitis in parent or sibling; maternal age > 18 years at the time of study enrollment. / Maternal HIV infection at time of delivery, plans for the family to move, does not speak English, or current use of progesterone to prevent preterm birth. | 20 |
| 2016 | Maternal and Developmental Risks from Environmental and Social Stressors (MADREs) | Pregnant women recruited from 3 OB/GYN clinics in Los Angeles, CA and their offspring. | To investigate the cumulative impact of prenatal and early life chemical contaminant and psychosocial stress exposure on early childhood obesity and metabolic health. | Pregnant women <30 weeks gestation; Singleton pregnancy; >18 years of age; HIV negative; Not currently incarcerated; No known physical, cognitive or mental disability that could prohibit participation/informed consent. / Current incarceration: Known physical, cognitive or mental disability that would prohibit informed consent; HIV positive status; <18 years of age; Multiple pregnancy; >30 weeks gestation | 46 |
| 2000 | Pittsburgh Girls Study (PGS) | A longitudinal study, sampled from the population of the City of Pittsburgh (disadvantaged neighborhoods were over-sampled). | To examine behavioral and emotional development in a population-based sample of Black and White girls living in an urban environment. | Participation in Pittsburgh Girls Study, Wave 1, which required: Being between the ages of 5–8 in 2000-2001; Families living in the City of Pittsburgh. / Severe developmental delay or hearing impaired with no sign language skills; Mother unable to speak English | <5 |
| 2009 | New Hampshire Birth Cohort Study (NHBCS) | Pregnant women recruited from 5 prenatal clinics in the rural New Hampshire and their offspring. | To investigate the impact of in-utero and early life environmental contaminants (e.g., arsenic) on maternal prenatal health, birth outcomes, growth, and neurodevelopment. | 18-45 years; Household has private well; English speaking, mentally competent. / Multiple births | 47 |
| 2016 | Rochester | Women recruited during the first trimester of pregnancy from clinics in Rochester, NY serving high psychosocial risk populations, oversampled for psychosocial stress. | To examine if and how prenatal exposures “program” adaptive biological responses in the fetus and child and carry-forward effects on neurodevelopmental health and obesity, with a focus on inflammation as a biological mechanistic pathway. | 18-40 years; <13 wks gestation at start; medically normal risk, singleton pregnancy; no history of psychotic illness; ability to communicate in English. / Presence of significant immunological, endocrinological or other significant medical condition; <18 yrs | 59 |
| 2013 | Fair Start | Dominican or African American low-income mothers and their offspring recruited at OB/GYN clinics in New York, NY. | To investigate the associations between prenatal exposure to environmental toxic pollutants and adverse childhood outcomes, including reduced IQ, ADHD, and obesity. | Enrollment at the Columbia Center for Family and Community Medicine clinic. / Pregnant with more than one baby | 6 |
| 2013 | Illinois Kids Development Study (IKIDS) | Pregnant women recruited from 2 OB/GYN clinics in southern Illinois and their offspring. | To examine the unique and combined impact of prenatal exposure to endocrine disrupting chemicals and maternal stress on child neurodevelopmental health and functioning. | Pregnant women must be between 18-40 years of age; Fluent in English; Live within 30-minute drive from research lab; Prenatal care must be done at Christie or Carle OB-GYN clinics in Champaign-Urbana area; Birth of infant must happen at Presence Medical Center or Carle Foundation Hospital in Urbana; Only one child per mother may participate; Able to enroll in study and provide first urine sample by 14 weeks gestation; Able/willing to come to research lab for child assessments post-delivery. / High-risk pregnancy (including expecting multiple births); A serious health condition involving either the child or mother during pregnancy; A health condition that prevents or limits the child's participation in postnatal assessments; Mother loses custody of child and/or is unwilling or unable to complete postnatal surveys | 22 |
| 2014 | Chemicals in our Bodies (CIOB) | Pregnant women recruited from 2 hospitals in San Francisco, CA and their offspring. | To examine the unique and combined impact of prenatal exposure to endocrine disrupting chemicals and maternal stress on child neurodevelopmental health and functioning. | Pregnant women between 13- and 27-weeks gestation; Age 18+; English or Spanish speaking; Zuckerberg San Francisco General Hospital or the UCSF Betty Irene Moore Women's Hospital at Mission; Singleton births. / High risk pregnancy | 30 |
